# Molecular Mechanisms Underlying the Generation of Absence Seizures: Identification of Potential Targets for Therapeutic Intervention

**DOI:** 10.3390/ijms25189821

**Published:** 2024-09-11

**Authors:** Beulah Leitch

**Affiliations:** Department of Anatomy, School of Biomedical Sciences, Brain Health Research Centre, University of Otago, Dunedin 9054, New Zealand; beulah.leitch@otago.ac.nz

**Keywords:** thalamocortical network, feedforward inhibition, parvalbumin interneurons, AMPA receptors, GABA, somatosensory cortex, thalamus, reticular thalamic nucleus, striatum, cerebellum, genetic generalized epilepsies, absence seizures, spike-wave discharges, calcium channels, channelopathies, antiseizure medications, personalized medicines

## Abstract

Understanding the molecular mechanisms underlying the generation of absence seizures is crucial for developing effective, patient-specific treatments for childhood absence epilepsy (CAE). Currently, one-third of patients remain refractive to the antiseizure medications (ASMs), previously called antiepileptic drugs (AEDs), available to treat CAE. Additionally, these ASMs often produce serious side effects and can even exacerbate symptoms in some patients. Determining the precise cellular and molecular mechanisms directly responsible for causing this type of epilepsy has proven challenging as they appear to be complex and multifactorial in patients with different genetic backgrounds. Aberrant neuronal activity in CAE may be caused by several mechanisms that are not fully understood. Thus, dissecting the causal factors that could be targeted in the development of precision medicines without side effects remains a high priority and the ultimate goal in this field of epilepsy research. The aim of this review is to highlight our current understanding of potential causative mechanisms for absence seizure generation, based on the latest research using cutting-edge technologies. This information will be important for identifying potential targets for future therapeutic intervention.

## 1. Introduction

Epilepsy is a group of neurological disorders characterised by recurrent, unprovoked epileptic seizures. Epilepsy affects over 70 million people worldwide [1] and it is estimated that about 1 in 26 people will develop epilepsy at some point during their lifetime [2]. Globally, idiopathic epilepsy is ranked fifth among neurological disorders after stroke, migraine, dementia and meningitis [3]. An epileptic seizure is defined as “*a transient occurrence of signs and/or symptoms due to abnormal synchronous neuronal activity in the brain*” [4]. The International League Against Epilepsy (ILAE) in 2017 revised its classification of seizures [5,6] and epilepsies [7] using a multilevel classification framework involving three levels: 1. Seizure types, 2. Epilepsy types, and 3. Epilepsy syndromes (see Figure 1 in ref. [7]). The new classification was designed to improve patient categorisation in different clinical environments and to help in diagnosis and treatment.

The ILAE (2017) classification of seizure types (Level 1, Figure 1 in ref. [7]) comprises focal onset, generalised onset, and unknown onset. Seizures with a “focal onset” originate within brain networks limited to one hemisphere and can be discretely localised or more widely distributed. Seizures with a “generalised onset” involve both hemispheres. Although they may originate at a particular initiation site within the brain (see Section 3.1), they rapidly engage bilaterally distributed networks, which can include cortical and subcortical structures but not necessarily the entire cortex. Generalised seizures can range from tonic–clonic seizures (during which the patient’s body and limbs becomes stiff followed by rhythmic jerking or twitching and loss of consciousness) to absence seizures where the patient only appears briefly to be unaware or “absent” (see Section 2.1). A classification of “unknown onset” is used where the patient has been diagnosed with epilepsy but there is insufficient information available to differentiate focal or generalised onset.

A diagnosis of “generalised” as the Epilepsy type (Level 2, Figure 1 in ref. [7]), is made on clinical grounds supported by findings from electroencephalography (EEG) showing generalised spike and wave discharges (SWDs). Generalised epilepsies include the subgroup of idiopathic generalised epilepsies (IGEs), which now fall within the group of genetic generalised epilepsies (GGEs) where there is evidence of a genetic aetiology (but note that “genetic” in this context is not synonymous with inherited; see [7,8] for the full ILAE description of GGEs).

Epilepsy syndromes (Level 3, Figure 1 in ref. [7]) apply to epilepsies with a cluster of shared features. Four well-established IGEs (i.e., epilepsies characterised by an underlying genetic predisposition) fall within this category: childhood absence epilepsy (CAE); juvenile absence epilepsy (JAE); juvenile myoclonic epilepsy (JME); and epilepsy with generalised tonic-clonic seizures alone (GTCS). Hirsch et al. [8] further defined these four epilepsies based on updated diagnostic criteria (determined by the ILAE’s Task Force 2017–2021 panel of experts). The updated set of criteria incorporated information from recent advances in genetic, imaging and EEG studies. The four epilepsy syndromes (CAE, JAE, JME, and GTCS) have now been identified as a special grouping of IGEs among GGEs within the current classification of seizures and epilepsies. They have polygenic inheritance with or without environmental contributory factors [8].

In this review, the focus is on highlighting our current understanding of potential causative mechanisms for absence seizure generation in CAE based on evidence from the latest research using cutting-edge technologies. The aetiology of absence seizures is poorly understood due to genetic and phenotypic heterogeneity. The main aim is to critically review the literature that has contributed to unravelling causative molecular mechanisms underpinning absence seizures genesis and thereby identify potential targets for future therapeutic intervention. This review begins by outlining the classification of absence seizures and CAE (Section 2). It then describes the cortico-thalamo-cortical (CTC) network where absence seizures are known to arise and considers cellular and molecular mechanisms that could underlie the generation of absence seizures in thalamic and cortical microcircuits (Section 3). The potential roles of the basal ganglia and cerebellum in networks underlying epileptogenesis are also discussed (Section 4). Underlying causal genetic and molecular mechanisms are then critically analysed with a view to identifying potential targets for development of future precision medicines (Section 5). Current therapies are reviewed and directions for future therapeutic strategies are proposed (Section 6). Finally, research still needed to be undertaken is briefly outlined and conclusions are drawn (Section 7).

## 2. Absence Seizures and Genetic Generalised Epilepsies

### 2.1. ILAE Classification and Diagnosis of Absence Seizures

Typical absence seizures are a type of generalised, nonmotor seizure characterised by a sudden, brief loss of consciousness and responsiveness termed an “absence”. They have been categorised as “nonmotor” by the ILAE 2017 [5,6,7] (but also see [9,10] for an alternative viewpoint challenging this classification). Generalised seizures involve hyperexcitation within neural networks in each hemisphere. Absence seizures involve pathological hypersynchronous oscillatory firing of neurons in the cortico-thalamo-cortical (CTC) network on both sides of the brain (see Section 3). EEG is crucial for diagnosing typical absence seizures. The hallmark signature on EEG is a generalised 2.5–4 Hz SWD accompanied by behavioural arrest. Although arrested behaviour is a characteristic of absences, automatisms such as lip smacking, blinking, or slight movements of the hands may occur in some children during the seizure [11]. It should also be noted that in practice, the electrographic presentation of SWDs can be highly variable even in the same patient.

### 2.2. Genetic Factors and the Classification of Absence Seizures

Epilepsies characterised by typical absence seizures are now known to be genetic in origin (see ILAE position statement [8] for full definition). However, the exact underlying cause(s) can vary among individuals, depending on their genetic background (for reviews, see [12,13,14,15]). The ILAE 2017 designated epilepsies within the “generalised epilepsy” group that display generalised SWDs to a genetic aetiology based on clinical genetic studies such as human twin studies and family research study data. Monozygotic twin studies show 70% concordance for generalised SWDs and absence seizures [16,17,18,19]. CAE falls within the subgroup of IGE syndromes, which have polygenic inheritance factors [20]; however, only a few genes conferring monogenic risk for CAE have been identified mainly through family studies [21,22,23,24]. It is important to note that most patients with IGE do not have a family history of epilepsy; these IGEs can arise as de novo mutations, or through complex inheritance. Hence, in the context of ILAE 2017 classification of these types of epilepsies, the term “genetic” refers to the cause and does not infer inherited.

### 2.3. Absence Seizures in Childhood Absence Epilepsy

CAE is the most common form of paediatric epilepsy. It affects 10–17% of all children with epilepsy. Typical absence seizures manifest as brief lapses in consciousness and mostly affect children between the ages of 4 and 14 years; they often stop spontaneously at around the time of puberty, although they can continue into adulthood. In young schoolchildren, these seizures may be mistaken for daydreaming or inattention due to their subtle nature. The onset of an absence seizure is often abrupt. Typically, the child will suddenly stop the activity they have been engaged in and stare blankly into space. After an absence seizure ends, the child resumes normal activity without any memory of the event (e.g., if the child has been counting just before the seizure started, they will stop during the seizure then resume counting from where they left off, after the seizure ends). The duration of a typical absence seizure is usually brief, lasting only a few seconds to half a minute. However, episodes can occur over a hundred times during a single day and so can severely disrupt daily activities and significantly impact the child’s cognitive development, academic performance, and social interactions.

Formerly, absence seizures were known as “*petit mal*” seizures and thought to be relatively benign due to their nonmotor presentation and short duration in contrast to the dramatic, violent muscle contractions and loss of consciousness evident during a generalised tonic-clonic (“*grand mal*”) seizure. However, this traditional view has been revised by more recent research, which has shown that absence seizures can lead to changes in brain morphology, including alterations in cortical thickness, depth of sulci, myelination, and connectivity, which may reflect aberrant neurodevelopmental processes, and/or adaptive responses to ongoing seizure activity [25,26,27,28,29,30,31,32]. Furthermore, cognitive deficits have also been identified in some patients with absence seizures.

Additionally, absence seizures are associated with comorbidities including behavioural and psychiatric disorders such as attention-deficit/hyperactivity disorder (ADHD), anxiety disorders, and depression [33]. Some children with absence epilepsy may have delays in their development of speech and language, motor skills, and social interaction. Furthermore, children with CAE are at greater risk of injuries from seizure-related accidents due to impaired awareness (i.e., consciousness). These could even be life-threatening under certain situations, for example, if they occurred while the child was swimming or cycling unsupervised. Therefore, early recognition, accurate diagnosis, and timely intervention with appropriate treatment and management strategies are essential for optimising outcomes and improving the quality of life of children affected by absence epilepsy. Seizure control and management of associated comorbidities, such as cognitive impairments or psychiatric disorders, are key goals in treating CAE. Educating carers, peers, and educators about this type of epilepsy and providing psychosocial support can help mitigate social and behavioural challenges, including social isolation and stigmatisation.

### 2.4. Changes in Brain Morphology Associated with Absence Seizures

Surface-based morphometry at high spatial resolution has been used to investigate the effects of CAE on cortical morphology and cognitive ability [31]. Tosun et al. [31] found that children with CAE do not demonstrate the normal age-related brain changes such as decreased cortical thickness and increased sulcal depth. They also found that children with CAE used different brain regions to perform cognitive functions compared to healthy controls. More recently, Kim et al. [34] analysed surface and volumetric magnetic resonance imaging (MRI) data from newly diagnosed CAE patients. They found a bilateral reduction in the frontotemporal cortical grey matter volume and an increase in the posterior medial cortical thickness, which were associated with the default mode network (DMN) in the brains of these children. Morphological alterations associated with absence epilepsy can vary among individuals and may be influenced by factors such as age of onset, seizure frequency, treatment history, and genetic predisposition. Collectively, the demonstration of morphometric abnormalities in children with CAE accompanied by cognitive impairments has led to a rejection of the previous traditional concept that CAE is a benign disorder [35].

### 2.5. Current Drug Treatments for Absence Seizures

Absence seizures are treated mainly with three antiseizure medications (ASMs): ethosuximide, valproate, and lamotrigine [36] (see Section 7). Ethosuximide is still the first-line drug of choice used to treat CAE despite having been developed over 50 years ago; it is the only AED to be used exclusively for absence seizures. Ethosuximide and valproate are broad-spectrum ASMs, which inhibit T-type calcium channels that control the firing rate of brain cells, reducing pathological oscillations and SWDs in CTC networks (see Section 6). However, as with other epilepsies, approximately 30% of CAE patients are refractory to ASMs used to treat their seizures or suffer severe side effects. Furthermore, some ASMs can exacerbate absence seizures. Recent studies indicate that there may be a genetic basis for the different responses between patients to current ASMs [37]. Further research is needed to identify the specific genes involved in CAE and to determine other factors that may play a role. Deciphering the specific underlying causative molecular and cellular mechanisms responsible for the initiation and generation of absence seizures is imperative in identifying specific targets for development of precision medicines. Section 3 discusses absence seizure initiation and propagation within the CTC network and unravels potential underlying causative mechanisms at the molecular and cellular level gleaned from recent studies using the latest optogenetic and chemogenetic technology [38,39].

## 3. Absence Seizures and Cortico-Thalamo-Cortical Networks

### 3.1. Cortical Site of Initiation of SWDs

Absence seizures are characterised by highly synchronised, generalised, pathological oscillations involving CTC networks on both sides of the brain (for a recent review, see [40]). There has been a long-running debate as to the relative importance of the cortex and thalamus in initiation, generation, propagation, and maintenance of absence seizures [41,42]. However, it is now generally accepted that SWDs are initiated in the somatosensory cortex before rapidly spreading to engage the CTC network in both hemispheres [43,44,45,46,47].

Studies using rodent genetic models of absence epilepsy have been instrumental in deciphering the site of initiation and the circuitry underpinning the genesis of absence seizures. Meeren et al. [43] demonstrated that absence seizure initiation begins within the perioral region of the primary somatosensory cortex by recording field potentials from multiple cortical and thalamic sites in freely moving Wistar Albino Glaxo from Rijswijk (WAG/Rij) rats during spontaneous SWDs. They showed that a cortical focus within the perioral region consistently led the thalamus during the first 500 ms of recording (see Figure 10 in ref. [43]). In another rodent model, the Genetic Absence Epilepsy Rats from Strasbourg (GAERS), paroxysmal activity was also found to originate in the primary somatosensory cortex [46,47]. Pollack et al. [46] found that epileptic discharges were initiated in layer 5/6 neurons of this cortical region, using in vivo intracellular recordings. Studer et al. [48] subsequently showed that SWDs were initiated in the barrel field primary somatosensory cortex that codes whisker-related information essential for rodents’ interactions with their environment.

In humans, noninvasive functional magnetic resonance imaging (fMRI) has been used to detect activity in specific areas of the brain during absence seizures, and to assess their relative contribution to any associated impairment in consciousness. Blood oxygenation level-dependent (BOLD) response is used in fMRI, as a surrogate for neuronal metabolic activity (see [49] for a review of the use of BOLD imaging in fMRI to detect physiological and pathological activity). Simultaneous EEG recordings of SWDs and fMRI show that absence seizures coincide with bilateral changes in the BOLD signal in several brain regions of children with absence epilepsy including the cortex, thalamus, subcortical structures, and areas of the DMN [50,51,52,53,54,55,56,57,58,59,60]. The magnitudes of fMRI changes in both the cortex and thalamus are related to the severity of impaired behavioural responses [54,57,58]. BOLD signals are mainly decreased in the cortex of patients during absence seizures and increased in the thalamus [50,55,57,61]. Thalamocortical activation is coincident with suspension of the default state of the brain [50,51]. Collectively, these data suggest that abnormal thalamocortical synchronisation during absence seizures results in momentary suspension of the DMN and widespread cortical deactivation, leading to a state of altered consciousness.

Interestingly, changes in BOLD signal amplitude and frequency have also been detected in specific cortical networks in children with absence seizures before the start of a seizure. Bai et al. [57] investigated the dynamic time course of typical childhood absence seizures in paediatric patients by simultaneously recording EEG and behavioural changes before, during, and after seizures, combined with fMRI imaging. They performed a direct analysis of the mean fMRI time course in the whole brain and in specific brain regions during the time period −20 s to +40 s relative to seizure onset. They found that changes in fMRI signal could be detected 8–14 s before behavioural changes. Small early fMRI increases were observed in the orbital/medial frontal and medial/lateral parietal cortex >5 s before seizure onset, followed by marked fMRI decreases continuing for >20 s after seizure end. Their analyses also confirmed early fMRI increases in several cortical areas, and relatively late increases in the thalamus.

Guo et al. [58] also used combined fMRI with EEG to investigate seizure activity during attention tasks. They found a spectrum of SWD features associated with different attention levels. Different absence seizures, characterised by small and large changes in BOLD signal amplitude and 2.5–4 Hz SWD on EEG, were associated with spared and impaired attention, respectively. They also investigated the temporal ordering of onset of BOLD activity in different brain regions related to generalised SWDs. BOLD amplitude changes were first evident in the DMN regions, including the medial frontal cortex; they were then detected in more extensive neocortical regions in frontal and parietal lobes (representing a task-positive network); finally, the thalamus, primary sensorimotor, and occipital regions showed BOLD amplitude changes.

More recently, Tangwiriyasakul et al. [61] reported increased phase synchrony of BOLD signals in sensorimotor cortical networks during non-ictal periods in patients with generalised SWDs. They used simultaneous EEG-fMRI to investigate whether generalised SWDs emerge during a dynamic evolution of brain network states in patients. They found that the generation of generalised SWDs involved a multistage process: a pro-ictal network state lasting at least 1 min, and a pre-ictal network state lasting several seconds prior to SWDs on EEG. They suggested that persistently high sensorimotor network synchrony, coupled with transiently low posterior cortical network synchrony, may be a state predisposing to generalised SWD onset.

Collectively, these studies indicate that there are both haemodynamic (BOLD) and EEG changes in cortical networks prior to generalised SWDs and absence seizures. Crunelli et al. [14] proposed that the pre-ictal changes in cortical areas, which occur prior to the engagement of CTC networks in both hemispheres, should be referred to as the cortical initiation network (CIN) to more accurately reflect the fact that they can occur at multiple cortical sites in humans and animal models prior to the development of generalised absence seizures. Furthermore, these authors advocate that the term “focus” should not be used in association with absence seizures to avoid confusion with “Focal Seizures”.

Overall, the consensus is that paroxysmal oscillation within corticothalamic loops is initiated in the CIN and that the large-scale synchronisation is mediated by way of an extremely fast intracortical spread of seizure activity. Thus, absence seizures are still considered to be correctly classified as a type of generalised seizure.

### 3.2. The Cortico-Thalamo-Cortical Network and Pathological SWD Oscillations

Although the site of initiation of electrographic activity for absence seizures is in the cortex, as described above, the full expression of the clinical symptoms associated with typical absence seizures requires interactions between cortical and thalamic networks [40,62]. It has even been proposed by some researchers that thalamic recruitment might be a key step necessary for the generalisation of absence seizures [14].

**Figure 1:**
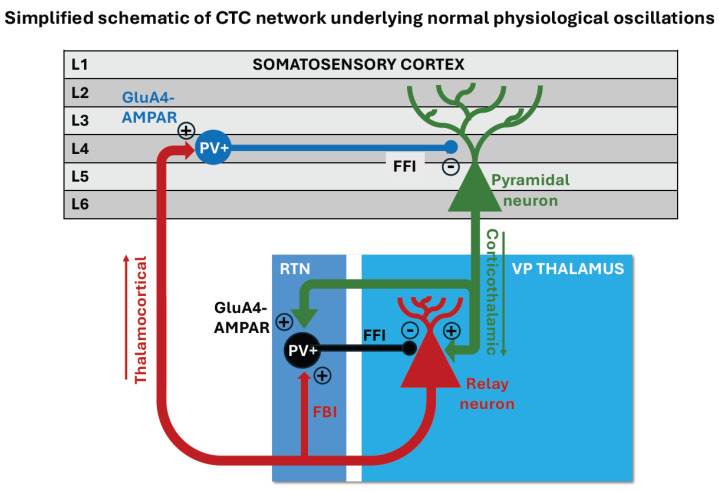
Simplified schematic of the normal cortico-thalamo-cortical (CTC) network in the rodent brain showing reciprocal connections between the pyramidal cells in the cortex (green triangles) and relay neurons in the VP thalamus (red triangles). PV+ inhibitory interneurons are strategically located to provide feedforward inhibition (FFI) to their target neurons. The PV+ inhibitory interneurons (black circles) in the reticular thalamic nucleus (RTN) project onto the relay neurons in the VP thalamus (black line) and provide fast feedforward inhibition (−). They are excited by corticothalamic projections onto their synapses (+), which contain GluA4-AMPA receptors. The CT-RTN pathway is stronger than the CT-VP relay neuron pathway. Normal physiological oscillations are generated when feedforward inhibition RTN-VP is followed by post-inhibitory rebound bursts of action potentials in VP relay neurons that in turn re-excite RTN interneurons and activate the feedback inhibitory pathway (FBI) from VP-RTN-VP. The PV+ inhibitory interneurons in the cortex (blue circle) are excited (+) by thalamocortical projections onto their synapses containing GluA4-AMPA receptors and provide fast feedforward inhibition (−) to pyramidal neurons (blue line).

The CTC network comprises reciprocal connections between the somatosensory cortex and the dorsal thalamus (see Figure 1 in ref. [40]). Corticothalamic (CT) axonal projections from pyramidal cells in layers 5/6 of the cortex make excitatory connections onto relay neurons in the ventroposterior (VP) thalamus, which in turn send thalamocortical (TC) axonal projections back to cortical neurons in layer 4 of the cortex [63,64,65]. The relay neurons in the VP thalamus also receive feedforward inhibition from inhibitory interneurons in the reticular thalamic nucleus (RTN), which forms a thin shell around the dorsal thalamus (Figure 1). These RTN inhibitory interneurons receive excitatory inputs via collaterals from both the CT and TC projections. The CT axon collaterals that target the inhibitory neuronal populations within the RTN thalamus provide a stronger synaptic pathway than the direct CT projections to relay neurons [66]. This enables cortical projections to dynamically regulate the balance of excitation and inhibition among thalamic neurons. The firing of the CTC network of neurons leads to normal physiological oscillations, which are involved in processing of sensory information. The dorsal thalamus acts as a relay station for all incoming sensory input from the periphery. It provides the major ascending input to the cortex. Thalamocortical oscillations are involved in arousal state, attention, and sleep.

Absence seizures arise from hypersynchronous pathological oscillations within the CTC network. Alterations in the interplay between excitatory and inhibitory neurons in cortical and thalamic microcircuits can generate the pathological rhythmic activity seen in SWDs [66]. This hypersynchronous, oscillatory activity is crucial for the propagation of absence seizures. However, the precise cellular and molecular events that transform normal physiological oscillation within the CTC network into pathological SWD oscillations are still under investigation and appear to be multifactorial.

Rodent models of absence epilepsy have proven essential to research the underlying cellular and molecular mechanisms for SWD generation, despite differences in their SWD frequency and developmental time course for the onset and termination of absence seizures, compared to human patients. In mouse and rat models of absence epilepsy, the EEG hallmark SWDs occur at 5–7 Hz and 7–11 Hz, respectively, whereas in humans, SWDs are slower, usually 2.5–4 Hz. In monogenic mouse models, the age of onset of absence seizures (second postnatal week) is developmentally equivalent to the age of onset in humans (typically 4 years). However, in mouse models, seizures persist into adulthood, unlike CAE, where absence epilepsy remits in 60% of children by early adolescence. Rat models, which are polygenic, only display seizures in adulthood. However, GAERS rats show SWD-associated changes in fMRI BOLD responses that mirror those seen in human BOLD studies [67] when tests are conducted on awake, non-anaesthetised animals. Awake GAERS exhibit decreased BOLD signals in the primary somatosensory cortex and increased signals in the VP thalamus during absence seizures, similar to humans.

#### 3.2.1. Monogenic Mouse Models of Absence Epilepsy

Monogenic mouse models of absence epilepsy provide a unique opportunity to investigate causal links between a singular defective molecular gene product and the mechanisms underlying pathological SWD oscillations within thalamocortical microcircuits. Unlike other forms of epilepsy, only genes related to ion channels have so far been linked to absence phenotypes. For a comprehensive review of the different monogenic mouse models of absence seizures that have been used to elucidate the mechanisms of epileptogenesis in absence epilepsy, see [68]. These monogenic mouse models all carry genetic mutations that consistently result in the typical phenotypic characteristics of absence epilepsy, i.e., SWDs accompanied by behavioural arrest.

**Figure 2:**
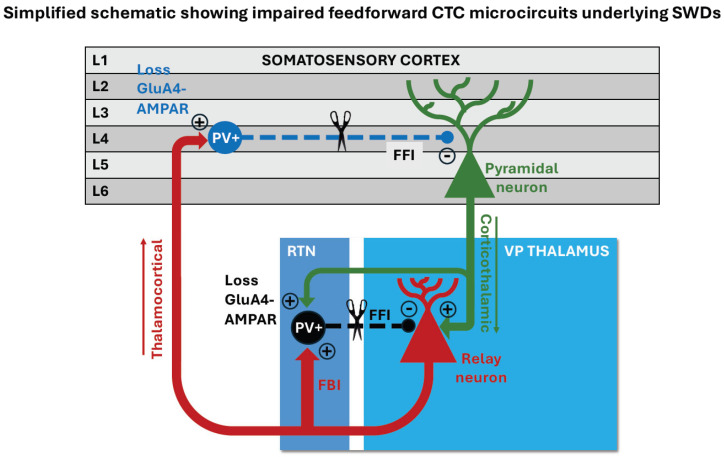
Simplified schematic showing impaired feedforward microcircuits in the cortico-thalamocortical (CTC) network in the rodent brain. Loss of GluA4-AMPA receptors at PV+ interneurons synapses in the stargazer mouse model and the GRIA4 knockout mouse results in reduced activation of the inhibitory PV+ interneurons and thus impaired feedforward inhibition in CTC microcircuits leading to disinhibition (dashed lines and scissors symbol) and absence seizures. Impaired feedforward inhibition, through loss of excitation onto inhibitory neurons, may be one underlying mechanism for SWDs in a subpopulation of patients with absence epilepsy.

Studies using the stargazer mouse model of absence epilepsy [69,70] have been particularly fruitful in revealing changes in cellular and molecular signalling pathways within the CTC network that are linked to absence seizure generation. The stargazer mouse has a spontaneous mutation in the voltage-dependent calcium channel γ2 subunit (*CACNG2*) gene [71], which severely reduces the expression of the protein stargazin and leads to deficits in alpha-amino-3-hydroxy-5-methyl-4-isoxazolepropionic acid (AMPA) receptors at excitatory synapses [72]. Stargazin is a member of the family of transmembrane AMPA receptor regulatory proteins (TARPs) that are essential for the proper trafficking and function of AMPA receptors. They are differentially expressed in specific neurons and brain regions [73,74,75]. Loss of stargazin (TARP-γ2) in the stargazer mutant mouse leads to absence epilepsy and cerebellar ataxia as a result of specific AMPA receptor deficits in the CTC network and cerebellum, respectively [69,70,76,77,78,79,80,81]. In the CTC network, the stargazin mutation leads to a specific loss of functional AMPA receptors at cortico-RTN synapses in the thalamus, and at synapses between TC projections and parvalbumin expressing (PV+) interneurons in the somatosensory cortex [62,78,79,80,81]. The specific loss of functional AMPA receptors at excitatory synapses in PV+ inhibitory interneurons in the cortex and RTN thalamus results in reduced excitation of fast-spiking inhibitory interneurons in CTC microcircuits, ultimately leading to SWDs on the EEG and behavioural arrest. Thus, disrupted AMPA receptor signalling in fast-feedforward interneurons is implicated in the pathogenesis of absence seizures (Figure 2).

Although mutations in *CACNG2* have not been identified in humans with absence epilepsy, several human genetic analyses suggest that TARPs can underlie familial epilepsy syndromes (for review, see [82]). Linkage and mutational analysis studies have shown a link between the *CACNG3* mutation and CAE [83]. Homozygosity mapping has also identified a 4 cM region within 22q13.1, which contains the *CACNG2* (human stargazin) locus in a family with epilepsy [84].

Further evidence that fast-feedforward disinhibition is a common mechanism that can lead to SWDs and absence seizures is provide by data from the *GRIA4* knockout mouse [85], which lacks the GluA4 AMPA receptor subunit. GluA4 is the most abundant subunit in RTN interneurons [86]. Loss of GluA4 in the *GRIA4* knockout results in a selective impairment at CT-RTN synapses, but not at CT-relay or relay neuron-RTN (feedback) synapses [66]. Hence, there is a specific impairment of thalamic feedforward inhibition from RTN interneurons to relay neurons in the VP thalamus due to weakened AMPA receptor-mediated excitatory input to inhibitory RTN interneurons and a strengthening of the CT-relay neuron pathway (Figure 2).

Another monogenic mouse model, which exhibits SWDs and absence seizures accompanied by behavioural arrest as a result of impaired fast-spiking interneurons, is the tottering mouse model [87]. Feedforward inhibition in the somatosensory cortex is markedly compromised and correlated to the developmental onset of absence seizures in the tottering mouse model [88]. These mice carry a spontaneous mutation in the *CACNA1A* gene leading to impaired release of the neurotransmitter gamma-aminobutyric acid (GABA) from fast-spiking inhibitory interneurons. The gene product affected is the α1 subunit of the P/Q-type calcium channel, causing reduced current in this high-voltage activated calcium channel [89]. *CACNA1A* mutations have subsequently been identified, through human genetic studies in cases of CAE [90].

Definitive evidence that disinhibition within the CTC network can lead to the generation of SWDs and absence seizures has been provided using cutting-edge Designer Receptor Exclusively Activated by Designer Drug (DREADD) technology to selectively silence feedforward inhibitory interneurons in either the cortex or thalamus of nonepileptic mice [91]. DREADDs are engineered G-protein coupled receptors [39]. Gi-DREADDs signal through the Gαi/o G-protein and inhibit neuronal signalling by inhibiting adenylate cyclase and downstream cAMP production; Gq-DREADDs signal through the Gαq/11 G-protein and activate neuronal firing through stimulating phospholipase C, which releases intracellular calcium stores. Focally silencing PV+ interneurons in the CTC network using inhibitory Gi-DREADDs induced SWD-like oscillations in EEG, which were associated with behavioural arrest and absence seizures [91]. Conversely, selectively activating feedforward interneurons in CTC microcircuits during pentylenetetrazol (PTZ)-induced seizures, using excitatory Gq-DREADDs, either prevented SWDs and absence seizures occurring or suppressed their severity [92].

#### 3.2.2. Polygenic Rat Models of Absence Epilepsy

Polygenic rat models of absence epilepsy, generated through inbreeding, have also provided important insights into the cellular and molecular mechanisms underlying pathological oscillatory discharges in the CTC network (for a detailed description of rat models of absence epilepsy, see [93,94,95]). In the GAERS and WAG/Rij rat models, absence seizure initiation is linked to enhanced excitability of pyramidal neurons in layer 5/6 of the somatosensory cortex [46,96]. However, mutual interactions between cortical and thalamic networks are required to generate the absence epilepsy phenotype.

Studies on GAERS rats have shown that enhanced GABA_A_ tonic inhibition in the VP thalamus contributes to the generation of SWD in the CTC network in this model [97,98]. The enhanced tonic inhibition is a consequence of higher levels of GABA transmitter in the VP thalamus [97] compared to the somatosensory cortex [99] due to a malfunction in the astrocytic GABA transporter GAT-1 (but not GAT-3) in the thalamus. GAT-1 is responsible for clearing GABA from the synaptic cleft after presynaptic release. Malfunctioning of this transport thus causes GABA levels to be elevated, leading to the activation of extrasynaptic GABA receptors on relay neurons and enhanced tonic inhibition. Enhanced tonic inhibition de-inactivates T-type calcium channels in VP relay neurons, which leads to reciprocal burst firing in thalamic and cortical excitatory neurons. The monogenic stargazer and lethargic mouse models also show reductions in the GAT-1 protein. However, in these mouse models, GAT-1 loss is a secondary effect to the primary disinhibition mechanism and is likely to be a compensatory homeostatic downregulation of GAT-1 in response to reduced GABA inhibition [100].

Simultaneous recordings of activity in cortical and thalamic neurons, in freely moving GAERS and WAG/Rij rats during absence seizures, have demonstrated that cortical afferent activity drives thalamic firing and controls the timing of the output from the thalamus via feedforward inhibition from RTN interneurons to the relay neurons [101]. More recent studies, using electrophysiological and imaging techniques with single-cell resolution in non-anaesthetised GAERS, have revealed four stable functional classes of cortical and thalamic firing during consciousness-impairing absence seizures [67,102]. Individual cortical and thalamic neurons express one of four distinct patterns of seizure-associated activity. The activity of cortical and thalamic neurons during absence seizures was predominantly characterised by a decreased, but highly synchronised, firing pattern. In all cases, changes in firing pattern preceded seizure onset by up 80 s. Irrespective of the type of firing, all cortical neurons displayed a marked increase in rhythmicity during SWDs compared to interictal periods.

Collectively, these data from rodent models of absence epilepsy indicate that there are multiple underlying cellular and molecular mechanisms that contribute to abnormal thalamocortical synchronisation during absence seizures. Identifying which specific ones are responsible for causing absence seizures in patients from different genetic backgrounds will be essential if precision medicines are to be developed that are tailored to an individual patient’s condition.

## 4. The Involvement of Other Brain Regions in Absence Seizures

### 4.1. The Role of the Striatum in Absence Seizures

It is generally accepted that absence seizures arise in the somatosensory cortex and thalamus (as described in Section 3). Nevertheless, there have been challenges to the long-held view that the cortico-thalamic circuit is the sole and exclusive causal source for absence epilepsy. A recent study demonstrated that absence epilepsy could be triggered by impaired cortico-striatal excitatory transmission [103]. Miyamoto et al. [103] created haplodeficient mice with mutations in *STXBP1* and *SCN2A* genes; these mutations are observed in patients with epilepsies. *STXBP1* encodes the presynaptic protein Munc18-1, which is essential for neurotransmitter release; *SCN2A* encodes a voltage-gated sodium channel α2 subunit (Nav1.2). Mice with haplodeficiencies for these genes exhibited absence seizures with SWDs, which were initiated by reduced cortical excitatory transmission into the striatum but not by impaired cortico-thalamic communication. Interestingly, there was a reduction in excitatory transmission from the neocortex to striatal fast-spiking interneurons in these mice. Activity in the fast-spiking interneurons in the striatum was transiently reduced at the onset of SWDs. Furthermore, SWDs could be suppressed by potentiation of AMPA receptors in the striatum but not the thalamus. Importantly, absence and “convulsive” seizures could be triggered, in a dose-dependent manner, by pharmacological inhibition of excitatory transmission in cortico-striatal fast-spiking interneurons. The authors concluded that impaired cortico-striatal excitatory transmission is a possible mechanism triggering epilepsy in *Stxbp1* and *Scn2a* haplodeficient mice [103]. They proposed that a pathologic decrease in cortico-striatal excitatory transmission onto fast-spiking interneurons due to genetic mutations represents a causal driver of SWDs linked to epileptogenic phenotypes.

These findings challenge previously held views that the basal ganglia (e.g., the striatum) is merely a modulator of SWDs primarily produced by thalamocortical circuits [104,105,106,107,108]. However, it should be noted that in other studies on mice, using either pharmacological suppression [109], cell ablation [110], or optogenetic suppression of striatal fast-spiking interneurons [111], neither SWDs nor epileptic seizures were reported. However, this may be due to the fact that EEG recordings needed to detect SWDs were not undertaken in these studies; SWDs and absence seizures can be difficult to detect in mice without EEG. Interestingly, fMRI studies in patients with IGE have demonstrated that in addition to the cortex, thalamus, and DMN, the caudate nuclei are also involved during generalised SWD paroxysms and absence seizures.

Miyamoto et al. [103] have proposed a novel cortico-striato-thalamic neural circuit model for absence epilepsy, implicating the basal ganglia via an indirect pathway. They propose that impaired cortico-striatal excitatory neurotransmission results in reduced activation of fast-spiking interneurons, which leads to the disinhibition of striatal medium spiny neurons (MSNs) in the caudate putamen. This leads to a sequence of events whereby activated MSNs over-suppress the globus pallidus externus (GPe), causing disinhibition of the subthalamic nucleus (STN), activation of the globus pallidus internus/substantia nigra pars reticulata (GPi/SNr), and over-suppression of the thalamus. As a consequence, thalamic relay neurons are hyperpolarised and produce rebound firing by de-inactivating Cav3.1 T-type calcium channels generating SWD and seizures. However, how the disinhibition of fast-spiking interneurons preferentially affects SWDs via the indirect pathway is still undetermined, as there are reciprocal connections between direct and indirect pathway neurons. [112].

### 4.2. The Role of the Cerebellum in Absence Seizures

While the cerebellum has not traditionally been associated with contributing to networks underlying epileptogenesis, recent human and animal studies indicate that the cerebellum can play a role in the emergence of epileptic seizures across a spectrum of seizure types, including absence seizures. In human imaging studies, structural and functional MRI has been used to detect activity in the different brain regions during seizures [113], and BOLD signal has been detected in the cerebellum of patients with CAE [54]. In animal studies, on-demand optogenetic and chemogenetic techniques have been used to interrogate cerebellar circuits in vivo [114,115].

#### 4.2.1. Evidence for Cerebellum Involvement in Epileptogenesis from Human Studies

Evidence for the involvement of the cerebellum in epilepsy has come from structural and functional MRI studies. Structural MRI data from large cohort human studies (e.g., global ENIGMA-Epilepsy study comprising 1602 adults with epilepsy and 1022 healthy controls across 22 sites [113]) have shown volume losses in the deep cerebellar and posterior lobe subregional grey matter in patients with chronic epilepsy. Other studies have demonstrated a reduction in cell number in the cerebellum linked to the occurrence of seizures in humans. The cerebellar cortex comprises three histological layers: an inner excitatory granular layer, a middle layer of inhibitory Purkinje cells (PCs), and an outer molecular layer. Significant reductions in PCs have been reported in the cerebellum of some epileptic patients [116,117]. PC loss is also linked to the occurrence of seizures in some rodent models [118]. The cerebellar GABAergic PCs represent the sole output from the cerebellum. They provide inhibitory output to the deep cerebellar nuclei [119]; thus, their specific loss in patients and rodent models of epilepsy suggests an association between cerebellar disinhibition and epileptogenesis [120].

Specific evidence for cerebellum involvement during absence seizures in humans has come from fMRI BOLD imaging in patients with CAE. These studies have demonstrated that during synchronised SWD activity in the thalamocortical network, there is BOLD activation in the thalamus, frontomesial cortex, and cerebellum, but BOLD deactivation in the DMN [121].

Electroencephalographic recordings and lesion studies have also provided evidence for the involvement of the cerebellum in human epileptogenesis. Cerebellar activity concomitant with seizures has been observed during recordings from the deep cerebellar nuclei of human patients with uncontrolled epileptic seizures [122]. Additionally, clinical studies have shown that cerebellar lesions can lead to cortical epileptiform activity and that complete surgical resection allows most patients to become free of seizures, whereas partial resection only achieves seizure-free status in under half of the patients [120]. Interestingly, targeting cerebellar structures using deep-brain stimulation produced therapeutic benefits for patients with seizures.

#### 4.2.2. Evidence for Cerebellum Involvement in Epileptogenesis from Animal Studies

Direct evidence of a correlation between SWDs in EEGs and neuronal activity in cerebellar cells has been provided by electrophysiological experiments in rodent models [123,124,125,126,127]. In the WAG/Rij rat model of absence epilepsy, SWD spindles recorded epidurally from the sensorimotor neocortex were correlated with single or multiple unit activity in the cerebellar cortex and deep cerebellar nuclei [123]. Phase-locked burst firing of cerebellar units during SWDs was independent of any rhythmic movement, thus confirming that cerebellar engagement was not just a consequence of motor aspects of seizures. Likewise, in the tottering mouse model of absence epilepsy, which has a mutation in the voltage-gated P/Q-type calcium channel (Cav2.1), the cerebellar PCs display rhythmic, synchronised firing during generalised SWDs [126,127]. Kros et al. [127] simultaneously recorded activity in the cerebral cortex and the cerebellum in awake tottering mice and demonstrated that simple spike firing in PCs was more synchronous during seizures. These researchers found that approximately half of the recorded PCs showed an ictal simple spike firing pattern that was phase-locked to SWDs. They also reported that 26% of PCs displayed increased complex spike activity and rhythmicity during SWDs. Seizure-related changes in PCs firing frequency, rhythmicity, and synchronicity were most prominent in the lateral cerebellum, which receives cerebral input via the inferior olive nuclei.

Further evidence that the cerebellum is implicated in generalised epileptic seizures has come from animal studies using on-demand optogenetic and chemogenetic techniques to interrogate cerebellar circuits in vivo [114,115,126]. In a recent optogenetic mouse study, where cerebellar input was shown to provide critical signals during generalised, “convulsive” motor seizures [115], virus tracing identified cerebellar and cerebral cortical afferents as robust contributors to the seizures. Microinfusion of lidocaine into the cerebellar nuclei (but not other brain regions) blocked seizure initiation, confirming that the cerebellum contributes substantially to the initiation of generalised seizures in this optogenetic mouse model. Kros et al. [126] used on-demand optogenetics to selectively activate cerebellar nuclear neurons during absence seizures in the tottering mouse model. They demonstrated that generalised SWDs could be effectively terminated in this rodent model at the onset of light delivery. Further proof that ongoing SWDs can be ceased by disrupting cerebellar nuclei rhythmic firing was achieved using a closed-loop channelrhodopsin-2 stimulation protocol. These chemogenetic and optogenetic studies indicate that deficits in P/Q-type calcium channels in cerebellar granule cells and PCs play an important role in epileptogenesis, and that precisely timed activation of the cerebellar nuclei neurons using these technologies can be used to stop ongoing SWDs. Data from rodent studies using new technologies, which allow precisely timed on-demand interrogation of specific cellular networks, indicate that rhythmic output from the cerebellum can contribute to the maintenance of generalised absence seizures and may even drive seizure activity in some cases.

Further evidence that abnormal activity in the cerebellum can directly contribute to causal mechanisms underlying absence seizures, in addition to being a consequence of epileptic activity, has been provided from restricted knockout of P/Q-type calcium channels in specific cerebellar cells. Genetic ablation of P/Q-type calcium channels in cerebellar granule cells (i.e., quirky knockout mouse model) or PCs (i.e., purky knockout mouse model) leads to recurrent SWDs [124,125]. Schwitalla et al. [114] showed that the incidence of SWDs could be decreased in both quirky and purky conditional knockout models by increasing the firing of their cerebellar nuclei activity via the activation of excitatory Gq-DREADDs. Conversely, decreasing cerebellar nuclei activity via inhibitory Gi-DREADDs caused an increase in the incidence of SWDs.

Collectively, these data provide evidence that the cerebellum is directly involved in the modulation of absence epilepsy and suggests that the cerebellum could be a potential therapeutic target for seizure control across a range of epilepsy types [120,128,129].

#### 4.2.3. Cerebellar Dysfunction and Ataxia in Animal Models with Absence Epilepsy

Several mouse models of absence epilepsy have cerebellar deficits in addition to absence seizures. For example, the mouse mutant models stargazer, ducky, lethargic, and tottering share phenotypic features, including ataxic gait and absence seizures with SWDs between 5 and 7 Hz [70,71]. In the stargazer model of absence epilepsy, cerebellar ataxia begins at around postnatal day 14 [70]. The cerebellar dysfunction in the stargazer mutant mouse is linked to impaired AMPA receptor expression and function at mossy fibre-granule cell synapses in the cerebellum [72,73,74,75,76,77] as a result of the mutation in the neuronal calcium channel γ subunit [70,71] which encodes TARP γ2 subunit stargazin [72,73,74,75]. Only the granule cells in the cerebellum depend exclusively on TARP γ2 (stargazin) to traffic AMPA receptors to their synapses; hence, AMPA receptor localisation is noticeably defective in these cells in the stargazer mouse. Stargazers also exhibit other cerebellar morphological changes associated with the mutation in stargazin, including reduced levels of GABA, changes in GRIP 1&2 scaffolding proteins, and calcium channel deficits [77,130,131,132,133].

Cerebellar dysfunction and ataxia have also been reported in patients with epilepsy [134]. There is a growing body of literature now implicating the cerebellum both indirectly and directly in human epileptogenesis. Evidence from both animal experiments and clinical epileptology suggests that the cerebellum has the ability to indirectly affect epileptic activity within the cerebral cortex. A direct role of the cerebellum in human epileptogenesis seems likely given the ability of cerebellar structures to generate epileptic activity, as described in the preceding sections. Attempts to control epilepsy by stimulating the cerebellar cortex and nuclei have already been attempted. Further research will be needed to confirm the precise role of the cerebellum in epileptogenesis in order to identify cerebellar structures to target for potential therapeutic interventions.

## 5. Identification of Causal Genetic and Molecular Mechanisms

### 5.1. Human Genome-Wide Association Studies

While clinical genetic studies, such as twin analyses, have shown that CAE has a strong genetic component, few human genes have been identified that confer a monogenic risk for CAE [8,21]. Identifying a specific causal link between a human gene mutation and absence seizures is inherently difficult because genome-wide association studies (GWAS) usually comprise human cohorts with a complex phenotype, which exhibits mixed epilepsies and other neurological deficits. However, recently, there has been some progress in identifying susceptibility variants through international research collaboration by large group consortia using genome-wide mega-analyses of GGEs.

The first GWAS of GGE syndromes [135], which included a large pure CAE cohort as part of a larger study group of over a thousand human subjects with common GGE syndromes, identified two susceptibility loci in chromosomal regions, 2p16.1 and 2q22.3. Later studies, undertaken by large ILAE consortia in 2014 [136] and in 2018 [137], identified 11 loci associated with the GGEs using genome-wide mega-analyses. These latter studies also confirmed 2p16.1 and 2q22.3 as significant loci. However, identification of the precise genes in these loci that are causally linked to the CAE phenotype is still under investigation. In a more recent study [138], which comprised CAE and JAE patients, lead associations were found for genes encoding ion channels *CACNA1G*, *EEF1A2*, *GABRG2*, and GABA_A_R. However, none of these genes achieved exome-wide significance. Furthermore, the human homologs of these genes are not located in the loci identified by GWAS as significant.

To date, most of the single gene mutations identified as being causally linked to absence seizures have come from studies on monogenic mouse models of absence epilepsy described in Section 3.2.1. These include single mutations in voltage-gated or ligand-gated channels in specific cortical or thalamic neuronal population and are discussed next in Section 5.2.

### 5.2. Genetic Mutations and Channelopathies Linked to Absence Epilepsy in Monogenic Mouse Models

Most of the mutated genes associated with absence epilepsy in rodent models encode ion channels. These include calcium channels (*CACNA1A*, *CACNA1G*, *CACNA1H*, *CACNA2D2*, *CACNB4*, *CACNG3*), sodium channels (*SCN8A*), potassium channels (*HCN2*), GABA receptor-related channels (*GABRA1*, *GABRB3*, *GABRG2*, *GAT-1*), and glutamate receptor-related channels (*GRIA4*, *CACNG2*, *CACNG2*, *CACNG4*, and *PICK1* (see Table 1 in ref. [68])). In most monogenic mouse models of absence epilepsy, seizures have only been detected following investigation into other neurological phenotypes when subsequent EEGs identified SWDs. In many mouse models, absence seizures are due to spontaneous mutations. In some cases, epileptic mice have been bred with targeted mutations in pathways implicated in IGEs, which reproduce the hallmark generalised SWDs and behavioural arrest characteristic of CAE.

#### 5.2.1. Calcium Channels

The *CACNA1A* gene encodes the P/Q-type (Cav2.1) calcium channel α1 subunit. The tottering mouse model of absence epilepsy carries a point mutation in the *CACNA1A* calcium channel [87] leading to impaired GABA release from fast-spiking interneurons and loss of feedforward inhibition. Selective deletion of *CACNA1A* in all cortical interneurons causes functional deficits in the fast-spiking, PV+ interneurons, resulting in a severe epilepsy phenotype involving both absence seizures and generalised tonic-clonic seizures [139]. However, the deletion of *CACNA1A* in excitatory cortical neurons causes a pure absence epilepsy phenotype in mice. Human *CACNA1A* mutation studies have also been identified in patients with CAE [90].

The *CACNA1G* and *CACNA1H* genes encode Cav3.1 and Cav3.2 T-type calcium channels, respectively. T-type calcium channels sustain post-inhibitory rebound bursts in thalamocortical neurons in the CTC network. They require membrane depolarisation to open and hyperpolarisation to recover. The *CACNA1G* (Cav3.1) type calcium channel is expressed predominantly in the cortex and thalamic relay nuclei in wild-type mice. Overexpression leads to a pure absence epilepsy phenotype [140]. Conversely, the deletion of functional α1G subunit-containing T-type calcium channels in relay neurons in the thalamus is protective against SWDs. Knockout of *CACNA1G* in epileptic stargazer, tottering, and lethargic mice eliminates SWD generation in these models of absence epilepsy [141]. On the other hand, *CACNA1G* knockout mice are resistant to gamma-hydroxybutyrate (GHB) and baclofen-induced absence seizures [112,142]. The *CACNA1H* (Cav3.2) type calcium channel is expressed predominantly in the RTN in the CTC network. The mutation is associated with absence seizures in the GAERS rat model [143]. GAERS rats have a gain of function mutation in *CACNA1H* (see [12,14] for comprehensive reviews of the genes and ion channels affected in polygenic rat models of absence epilepsy). Importantly, gene variants in *CACNA1G* and *CACNA1H* have been linked to human absence phenotypes [144,145,146]. Several human conditions, including epilepsy and its associated comorbidities, have been traced to variations in genes encoding T-type channels [147]. The AED ethosuximide, which is exclusively used for absence seizures, exerts its antiepileptic action by targeting T-type calcium channels (see Section 6).

Mutations in *CACNA2D2* and *CACNB4* encode P/Q-type calcium channel subunits α2δ-2 and β4, which are found in the ducky [148] and lethargic [149] mouse models of absence epilepsy, respectively. These subunits interact with the pore-forming α1 subunit of the P/Q-type calcium channel, leading to a loss of calcium current and, thus, impaired release of presynaptic neurotransmitter. Both of these mutations result in a phenotype resembling that of the tottering mouse *CACNA1A* mutation.

#### 5.2.2. GABA Receptor-Related Channels

Mutations in *GABRA1*, *GABRB3*, and *GABRG2* encode the GABA receptor channel subunits α1, β3, and γ2, respectively. The mouse models carrying these mutations [150,151,152,153] were only created after the mutation was first identified in CAE cohorts by human genetic linkage studies [21,154,155]. Mutations in *GABRA1* and *GABRG2* can lead to disinhibition in CTC networks and hyperexcitation of target excitatory neurons, e.g., layer 6 pyramidal cells in the cortex [156]. GABA receptor channels containing the α1subunit are primarily located at synaptic rather than extrasynaptic sites; hence, phasic rather than tonic inhibition is affected [157]. Mutations affecting the γ2 subunit, which mediates fast synaptic inhibition, lead to deficits in synaptic targeting of cortical GABA_A_ receptors and reduced GABA-gated chloride currents [153]. Mutations in *GABRB3* and *GAT-1* can lead to elevated levels of GABA neurotransmitter at synapses and enhanced tonic inhibition. The β3 subunit is specifically expressed in the RTN [158], and knockout of this subunit leads to increased synchrony in neuronal firing [159], which may be associated with increased tonic inhibition of thalamic relay neurons.

Changes in GABA_A_ receptor subunit expression have also been reported in the stargazer mouse model of absence epilepsy. In wild-type mice, GABA_A_ receptor α1 and β2 subunits are predominantly expressed in the VP region of the thalamus, whereas the α3 and β3 subunits are localised primarily in the RTN region. In adult stargazers, α1 and β2 subunit expression levels are significantly increased in the VP at inhibitory RTN-VP synapses, while α3 and β3 subunits in the RTN are unchanged [160,161]. However, these changes do not occur before seizure onset, which occurs at around postnatal day 16–18 in stargazers [162]. Likewise, changes in GABA_A_ receptor α1 subunit expression in the somatosensory cortex of the adult stargazer [163] are not evident in juvenile stargazers prior to seizure onset [164]. Hence, increased levels of GABA receptors at synapses in adult stargazers do not contribute directly to the onset of absence seizures. They are more likely a later consequence of seizure activity. The upregulation of GABA receptor subunits at targets of PV+ interneurons may be a mechanism to compensate for reduced feedforward inhibition in the stargazer CTC network. A partial increase in the expression of the GABA_A_ receptor α1 subunit has also been reported in the *GABRA1* heterozygous knockout mutant [151].

Although genes encoding GABA receptor subunits, including *GABRA1*, *GABRB3*, and *GABRG2*, have been associated with absence epilepsy, they have also been implicated in other forms of epilepsy, indicating that the GABA receptor dysfunction is not specifically linked to absence seizures.

#### 5.2.3. Glutamate Receptor-Related Channels

Mutations in *GRIA4*, *CACNG2* (stargazer mouse mutant) impact AMPA receptors, as already described in Section 3.2.1. The *GRIA4* knockout mouse and stargazer mutant mouse specifically lack calcium-permeable GluA4-containing AMPA receptors in fast spiking PV+ inhibitory interneurons in the RTN and cortex. Hence, there is a loss of feedforward inhibition in CTC microcircuits, leading to SWDs and absence seizures in these models (Figure 2).

From the data presented in this section, it is clear that several of the monogenic mutations discussed (*CACNA1A*, *GRIA4*, *GABARA1*) are associated with reduced feedforward inhibition in thalamic and cortical microcircuits, either through deficits in AMPA receptors at synapses in PV+ inhibitory interneurons or deficits in phasic GABA receptors on their targets. Hence, PV+ inhibitory interneurons could be potential targets to control SWDs using chemogenetic and optogenetic tools [91,92].

Collectively, studies on monogenic mouse models of absence epilepsy indicate that loss of feedforward inhibition in specific microcircuits is a common mechanism underlying absence seizure pathogenesis. These mouse models have been instrumental in identifying genetic mutations and channelopathies linked to absence epilepsy, which may be causal, and, thus, potential targets for future development of precision medicines. Developing effective therapies for absence epilepsy without unwanted side effects depends on understanding the exact causal mechanisms through which seizures are generated in individual patients. In the next section, current ASMs are critiqued, and future directions for developing more precisely targeted treatment options are considered.

## 6. Current Therapies and Future Directions

### 6.1. Current ASMs for Treating CAE and Their Limitations

The most commonly used first-line ASMs for treating CAE are ethosuximide, valproic acid, and lamotrigine. However, each of these drugs has various side effects [165]. Practical guidelines for their use in clinical practice [166] have been based on double-blind, randomised, controlled trials involving children with newly diagnosed and untreated CAE and follow-up studies [167,168,169]. Children aged between 4 and 10 years old were only included in these trials if they presented with absence seizures with no previous history of generalised tonic–clonic seizures [167]. Ethosuximide is still considered the optimal initial treatment for CAE [166] as it is associated with fewer adverse neuropsychological side effects on attention compared to the other ASMs. It is the only drug exclusively used to treat absence seizures (it has no effect on other types of seizure) and has been in clinical use since it was first introduced in 1958. Ethosuximide selectively blocks transient T-type calcium currents in thalamic neurons, which inhibit the thalamocortical circuits responsible for generating the SWDs underlying absence seizures. However, its efficacy as a T-type calcium channel blocker has been questioned [170].

Valproic acid is the preferred second AED for treatment of absence seizures if ethosuximide fails, whether due to reduced efficacy or intolerability reasons. It is a broad-spectrum antiepileptic medication and appears to have multiple mechanisms of action, including elevating GABA levels in the brain, blocking voltage-sensitive sodium channels, and activating calcium-dependent potassium conductance [171]. However, the specific mechanism by which valproic acid prevents absence seizures is unknown. Valproic acid has a greater number of potential adverse side effects than ethosuximide. It also has increased risks of teratogenicity compared to the other ASMs; neurodevelopmental impairments have also been reported after foetal exposure [172]. Hence, it is not used to treat absence seizures in adult pregnant females.

In clinical trials, ethosuximide and valproic acid were found to be more effective than lamotrigine for initial monotherapy of CAE [169]. However, lamotrigine may be used as a second monotherapy when ethosuximide has failed to be effective and where there are contraindications for the use of valproic acid for a specific patient. Its mode of action is not fully understood, but it is in part related to blocking of voltage-dependent sodium channels. By selectively binding to and inhibiting voltage-gated sodium channels, it stabilises presynaptic neuronal membranes and inhibits the presynaptic release of glutamate and aspartate. It has not been shown to have significant effects on other neurotransmitters such as serotonin, norepinephrine, or dopamine [173]. It has also been proposed that it acts by inhibition of Nav1.1 sodium channels on GABAergic neurons implicated in generalised epilepsies [174].

Monotherapies or combined treatments with ethosuximide, valproic acid, and lamotrigine effectively treat approximately two-thirds of patients suffering from CAE, and most achieve spontaneous remission in early adolescence. However, over 30% are refractive to these ASMs or suffer intolerable side effects. Some patients continue to require treatment into adulthood or develop other generalised epilepsy syndromes [175,176,177,178,179,180]. Why some patients tolerate current ASMs and other do not is poorly understood. Likewise, why remission occurs in some patients but not others is unclear. Both probably reflect a combination of factors including the individual patient’s genetic background.

A study by Glauser et al. [37] investigated whether there was a genetic basis for different responses to the three most commonly used drugs for CAE. The study was part of a multiconsortium (32-centre), randomised controlled clinical trial that compared the effects of these three ASMs in 446 children who were recently diagnosed with CAE. It focused on three genes that code for T-type calcium channels implicated in CAE and one gene that codes for a transporter that shuttles the drugs out of the brain. The study found that two specific forms of the calcium channel genes occurred more often in children for whom ethosuximide did not work. Two other variants of the calcium channel genes were found in children for whom lamotrigine did work. However, they also found a variant of the drug transporter gene, which was associated with a continuation of seizures. In vitro experiments on the calcium channel gene variant associated with ethosuximide failure in patients confirmed that the drug was less efficient in inhibiting these T-type calcium channels. Hence, the genetic form of a calcium channel may determine a patient’s response to these drugs. Knowledge of specific gene variants in children with CAE may help predict what drugs would work best for them. Furthermore, understanding the genetic factors underlying CAE and the causative mechanisms for the variability of responses between patients to ASM treatments will be important in designing future precision medicines that are more patient-specific and that have fewer side effects. The variability in patient responses to current ASMs highlights the need for more precision medicines that are tailored to an individual patient’s needs.

### 6.2. Development of Precision Medicines to Treat CAE

The development of precision medicines to treat CAE will require identification of cell-type-specific molecular targets that underpin the genesis of absence seizures in individual children. As our understanding of the aetiology and genetic basis of absence epilepsies grows, it may be possible to design new treatment options for patients with refractory epilepsies that do not respond to current ASMs.

In a recent review by Byrne et al. [181], the current and potential use of precision medicines to treat GGE was considered. These authors proposed a six-tier approach to defining precision therapeutics in epilepsy and considered how this could be incorporated into a clinical trial design. The six treatment tiers were defined by how precisely the mechanism of action addressed the aetiology. The lowest tiers represented treatment options based on historical responses of certain epilepsy phenotypes to specific medication. The highest tiers in their approach were speculative and looked to future treatment options with highly disease-specific therapies based on the correction of underlying genomic and proteomic issues. The top tier in their precision therapy model proposed future therapies, which would target genes and networks that rescue the whole phenotype. This would require an in-depth understanding of the complex interactions of gene products within regulatory networks and aberrant pathways, which allows precisely directed therapy and phenotype reversal. It is acknowledged that achieving more precise ways to control epilepsy in individuals will require in-depth, bioinformatic analyses of correlations between genotype and phenotype. This, in turn, will require establishing patient registries complete with accurate genetic information. However, there are likely to be other factors beyond genomics that must be considered in designing finely-tuned effective therapies that abolish seizures and attenuate the phenotype for each patient.

### 6.3. Future Treatment Strategies

Some researchers have suggested that there should be a shift in focus regarding when to treat children with absence seizures. Rather than using ASMs to treat absence seizures when they first appear, therapeutic approaches should be targeted at preventing seizures from occurring in the first place. Early intervention as a treatment strategy has been proposed based on recent findings from human and animal studies, which show that electrophysiological and behavioural changes occur before seizure initiation. McCafferty et al. [67] demonstrated, using fMRI and EEG, that prolonged brain state changes precede consciousness-impairing seizures in awake GAERS rats. Electrophysiological and behavioural changes were observed 40–60 s before obvious SWD onset. In human fMRI studies, pre-ictal changes occurred tens of seconds before SWDs on EEG [52,56,57,58]. Furthermore, early treatment with ethosuximide has been shown to impede the epileptogenesis of absence epilepsy in both humans [182] and rodents [183]. Chronic treatment with ethosuximide had disease-modifying effects, with antiepileptogenic effects against absence seizures and mitigation of behavioural comorbidities.

## 7. Conclusions

The majority of identified GGEs are due to altered ionic channel function or genes that play a role in cell excitability. Ionic channels are important in the development of the immature brain. Mutations causing prolonged activation or inactivation of channels during development may cause altered brain maturation, leading to epileptogenesis. Clinically relevant mutations to ion channels are routinely characterised as loss or gain of function mutations. However, some researchers consider that personalised medicine approaches based on the characterisation of loss or gain of function mutations will have limited therapeutic success, as translation of this information to neuronal firing for different neuronal cell types is currently not well understood [184]. Computational modelling and simulations have revealed that the effects of channelopathies on neuronal excitability are cell-type-dependent [184]. Determining cell-type-specific effects will be essential in developing precision ASMs and personalised medicine approaches.

The advent of new technologies to identify and analyse complex molecular information at single-cell resolution and the ability to manipulate cellular and neuronal networks with temporal and spatial precision using optogenetics and chemogenetics will greatly help in achieving the goal of identifying cell-type dependency of ion channel mutations on firing. Additionally, designer receptor-based technologies may someday become therapeutic options themselves, as the barriers in implementing these approaches in the clinic are overcome.

Further research is needed to decipher specific genotype–phenotype correlations to discern how a variant in a particular ion channel gene leads to the specific clinical presentation. A precise understanding of the functional consequences of most variants is still unknown. Detailed characterisation of variants would potentially allow personalised treatments to be designed [181,185]. The potential to use precision drugs to manipulate developmental plasticity to prevent the phenotypic appearance of inherited absence seizures is on the horizon.
